# Both 25-Hydroxyvitamin-D_3_ and 1,25-Dihydroxyvitamin-D_3_ Reduces Inflammatory Response in Human Periodontal Ligament Cells

**DOI:** 10.1371/journal.pone.0090301

**Published:** 2014-02-28

**Authors:** Oleh Andrukhov, Olena Andrukhova, Ulamnemekh Hulan, Yan Tang, Hans-Peter Bantleon, Xiaohui Rausch-Fan

**Affiliations:** 1 Division of Oral Biology, Bernhard Gottlieb School of Dentistry, Medical University of Vienna, Vienna, Austria; 2 Department of Biomedical Science, University of Veterinary Medicine, Vienna, Austria; 3 Department of Restorative Science, School of Dentistry, Health Science University of Mongolia, Ulan Bator, Mongolia; 4 Department of Stomatology, Xuanwu Hospital, Capital Medical University, Beijing, China; 5 Division of Orthodontics, Bernhard Gottlieb School of Dentistry, Medical University of Vienna, Vienna, Austria; Nihon University School of Medicine, Japan

## Abstract

Periodontitis is an inflammatory disease leading to the destruction of periodontal tissue. Vitamin D_3_ is an important hormone involved in the preservation of serum calcium and phosphate levels, regulation of bone metabolism and inflammatory response. Recent studies suggest that vitamin D_3_ metabolism might play a role in the progression of periodontitis. The aim of the present study was to examine the effects of 25(OH)D_3_, which is stable form of vitamin D_3_ in blood, and biologically active form 1,25(OH)_2_D_3_ on the production of interleukin-6 (IL-6), interleukin-8 (IL-8), and monocyte chemotactic protein-1 (MCP-1) by cells of periodontal ligament. Commercially available human periodontal ligament fibroblasts (hPdLF) and primary human periodontal ligament cells (hPdLC) were used. Cells were stimulated with either *Porphyromonas gingivalis* lipopolysaccharide (LPS) or heat-killed *P. ginigvalis* in the presence or in the absence of 25(OH)D_3_ or 1,25(OH)_2_D_3_ at concentrations of 10–100 nM. Stimulation of cells with either *P. gingivalis* LPS or heat-killed *P. gingivalis* resulted in a significant increase of the expression levels of IL-6, IL-8, and MCP-1 in gene as well as in protein levels, measured by qPCR and ELISA, respectively. The production of these pro-inflammatory mediators in hPdLF was significantly inhibited by both 25(OH)D_3_ and 1,25(OH)_2_D_3_ in a dose-dependent manner. In primary hPdLCs, both 25(OH)D_3_ and 1,25(OH)_2_D_3_ inhibited the production of IL-8 and MCP-1 but have no significant effect on the IL-6 production. The effect of both 25(OH)D_3_ and 1,25(OH)_2_D_3_ was abolished by specific knockdown of vitamin D_3_ receptor by siRNA. Our data suggest that vitamin D_3_ might play an important role in the modulation of periodontal inflammation via regulation of cytokine production by cells of periodontal ligament. Further studies are required for better understanding of the extents of this anti-inflammatory effect and its involvement in the progression of periodontal disease.

## Introduction

Vitamin D_3_ is known to play an important role in the bone metabolism and mineral homeostasis [1]. The major sources of vitamin D_3_ in organism are production by skin on the sun exposure and dietary supplements. To become metabolically active, vitamin D_3_ is first converted by liver to 25(OH)D_3_ (calcifediol), which has a half life time of about 15 day [2]. Calcifideol could be further converted into the active form of vitamin D_3_ calcitriol (1,25(OH)_2_D_3_) by specific enzyme 25(OH)D-1α-hydroxylase. The half life time of 1,25(OH)_2_D_3_ is about 15 h [2] and its biological effects are mediated by activation of the vitamin D_3_ receptor (VDR), a member of the nuclear receptor superfamily [3]. For a long time it was thought that the expression of 1α-hydroxylase is limited to kidney, but now this enzyme is also found to be expressed in numerous extrarenal tissues [4]. There are accumulating evidences that vitamin D_3_ is also involved in the regulation of immune response [5].

Periodontitis is a chronic bacterial infectious disease that affects tooth supporting tissues of periodontium [6], [7]. Periodontitis is caused by overgrow of some anaerobic Gram-negative bacteria, which trigger host responses causing most of the tissue damages, and might lead to substantial loss of alveolar bone and eventually the loss of teeth [8]. Especially “red complex bacteria” that include the periodontal pathogens *Porphyromonas gingivalis, Treponema denticola*, and *Tannerella forsythia* have been strongly associated with clinical measurements of periodontitis [9]. The association between vitamin D_3_ and periodontitis is currently under investigation and its role in the progression of periodontitis is not entirely understood [10]. Some studies report decreased serum levels of vitamin D_3_ in periodontitis as well as a negative correlation between serum vitamin D_3_ levels and severity of periodontal inflammation [11], [12]. In contrast, other studies show the increased serum levels of vitamin D_3_ in patients with aggressive periodontitis and positive association between serum vitamin D_3_ concentration and periodontal disease severity [13], [14]. Thus, the role of vitamin D_3_ in periodontal disease needs to be further investigated.

Periodontal ligament is a structure connecting teeth to the alveolar bone and seems to actively participate in alveolar bone remodelling [15]. Periodontal ligament cells (PDLs) are fibroblast-like cells characterized by collagen production but also possessing some osteoblastic features (for review, see [16]). In addition, periodontal ligament cells produce several pro-inflammatory mediators when stimulated with *Porphyromonas gingivalis* and/or its components [16], [17], [18]. Periodontal ligament cells isolated from *P. gingivalis*-positive periodontitis patients exhibit increased cytokine production in response to *P. gingivalis* [19]. Due to the proximity of periodontal ligament to alveolar bone, the cytokine production by periodontal ligament cells might influence the processes of bone resorption in periodontal disease. Vitamin D_3_ might have an important role in the function of periodontal ligament in periodontal disease, because periodontal ligament cells express 1α-hydroxylase and convert 25(OH)D_3_ into 1,25(OH)_2_D_3_ [20], [21]. A recent study shows that 1,25(OH)_2_D_3_ inhibits production of interleukin-8 by primary human periodontal ligament cells but has no effect on production of IL-6 [22]. Yet, it is not known if 25(OH)D_3_, which is a biological precursor of 1,25(OH)_2_D_3_ and main form of vitamin D_3_ in blood, could also influence the inflammatory response in cells of human periodontal ligament.

The main aim of the present study was to investigate if 25(OH)D_3_ as well as 1,25(OH)_2_D_3_ influence the production of pro-inflammatory mediators in cells of human periodontal ligament in response to stimulation with periodontal pathogens. To answer this question we investigated the effect of 25(OH)D_3_ and 1,25(OH)_2_D_3_ on the production of interleukin-6 (IL-6), interleukin-8 (IL-8), and monocyte chemoattractant protein 1 (MCP-1) by cells of human periodontal ligament in response to stimulation with *P. gingivalis* LPS or heat-killed *P. gingivalis*. The contribution of VDR on the effect of both vitamin D_3_ forms was investigated in the experiments with deletion of this protein using small interfering RNA (siRNA).

In the present study, we use commercially available primary human periodontal ligament fibroblasts (hPdLF), which represent standardized model of periodontal ligament cells. In addition, the effect of both vitamin D_3_ forms was investigated on the primary human periodontal ligament cells (hPdLC) isolated from six different donors. Our results revealed that both 25(OH)D_3_ and 1,25(OH)_2_D_3_ might modulate periodontal inflammation via regulation of cytokine production by cells of periodontal ligament.

## Materials And Methods

### Ethic Statement

Protocol for primary human periodontal ligament cells isolation was approved by the Ethics Committee of the Medical University of Vienna. Patients were informed in details before the surgical procedures and gave their written agreement.

### Cell Culture and reagents

Primary commercially available Clonetics human periodontal ligament fibroblasts (hPdLF) isolated from 16-year old male (Lonza, Switzerland) were used in the present study. These cells were shown to produce pro-inflammatory mediators and express osteogenesis-related genes, which is characteristic for periodontal ligament cells [23], [24], [25]. In addition, primary human periodontal ligament cells (hPdLC) were isolated from periodontally healthy donors undergoing routine extraction of their third molar teeth by outgrow method [26]. hPdLC were isolated by scraping the ligament tissue from the teeth root surface and cultured in Dulbecco's modified Eagle's medium (DMEM), supplemented with 10% fetal bovine serum (FBS), streptomycin (50 µg/ml) and penicillin (100 U/ml) under humidified air atmosphere of 5% CO_2_ at 37°C. Cells were cultured in Dulbecco's modified Eagle's medium (DMEM; Invitrogen), supplemented with 10% of FBS, 100 U/mL penicillin, and 100 µg/mL streptomycin at 37°C in a humidified atmosphere containing 5% CO_2_. Cells from passage levels 3–6 were used in this study.

Commercially available ultrapure *P. gingivalis* LPS, heat-killed *P. gingivalis*, and ultrapure *E. coli* LPS (all from Invivogen, San Diego, USA) were used in the present study. As reported by other study [27], LPS preparations are free from contaminating lipoproteins.

### Cells stimulation

Cells were seeded in a 24-well plate at a density of 5×10^4^ cells per well containing 0.5 mL of DMEM medium supplemented with 10% FBS. After 24 h, cells were stimulated with either *P. gingivalis* LPS (1 µg/ml) or heat-killed *P. gingivalis* (10^8^ cells/ml) in DMEM supplemented with 2% FBS. Stimulation was performed either in the presence or in the absence of 25(OH)D_3_ (10–100 nM, Cayman Chemicals, Ann Arbor, USA) or 1,25(OH)_2_D_3_ (10–100 nM, Sigma, San Diego, USA). Each experimental group included three wells. After stimulation for 24 h, the cellular mRNA expression levels of IL-6, IL-8 and MCP-1 in cells as well as the content of corresponding proteins in the conditioned media were determined.

### Quantitative PCR

The mRNA expression levels of IL-6, IL-8, and MCP-1 were determined by qPCR as described previously [28], [29], taking the β-actin encoding gene as internal reference. Isolation of mRNA and transcription into cDNA was performed using the TaqMan Gene Expression Cells-to-CT kit (Ambion/Applied Biosystems, Foster City, CA, USA) according to the manufacturer's instructions. This kit provides good accuracy and superior sensitivity of gene-expression analysis [30]. qPCR was performed on an ABI StepOnePlus device (Applied Biosystems) in paired reactions using the Taqman gene expression assays with following ID numbers (all from Applied Biosystems): IL-6, Hs00985639_m1; IL-8, Hs00174103_m1; MCP-1, Hs00234140_m1; β-actin, Hs99999903_m1. qPCR reactions were performed in triplicate in 96-well plates using the following thermocycling conditions: 95°C for 10 min; 40 cycles, each for 15 s at 95°C and at 60°C for 1 min. The point at which the PCR product was first detected above a fixed threshold (cycle threshold, C_t_), was determined for each sample. Changes in the expression of target genes were calculated using the 2^−▵▵Ct^ method, where ▵▵C_t_  =  (C_t_^target^−C_t_^β−actin^)_sample_−(C_t_^target^−C_t_^β−actin^)_control_, taking an untreated sample as a control.

### Measurements of cytokines in supernatants

Commercially available ELISA kits (Hoelzel Diagnostika, Cologne, Germany) were used for measurements of IL-6, IL-8, and MCP-1 levels in the conditioned media. For measurement of IL-6 and MCP-1 samples were not diluted, whereas for measurements of IL-8 samples were diluted 1∶10.

### RNA Interference of VDR

The highly specific technique of small interfering RNA (siRNA) was used to knockdown the expression of VDR in hPdLF. Cells were seeded at a density of 3×10^4^ cells per well containing 0.5 mL of DMEM medium supplemented with 10% FBS without antibiotics. 24 h later, the cells were transfected with either VDR siRNA (Cat. Nr. Sc-106692, Santa Cruz Biotechnology, Heidelberg, Germany) or a non-silencing control siRNA (Cat. Nr. Sc-37007, Santa Cruz Biotechnology) using siRNA Reagent System (Cat. Nr. Sc-45064, Santa Cruz Biotechnology) according to the manufacturers protocol. 48 h after transfection, cells were stimulated by *P. gingivalis* LPS or heat-killed *P. gingivalis* in the presence or in the absence of 25(OH)D_3_ (100 nM) or 1,25(OH)_2_D_3_ (100 nM). Stimulation was performed in DMEM containing 2% of FBS, 100 U/mL penicillin, and 100 µg/mL streptomycin. After 24 h, the gene expression levels of pro-inflammatory mediators IL-6, IL-8, and MCP-1 as well as the content of corresponding proteins in conditioned media were investigated. The effectivity of siRNA transfection was controlled by western blot. Protein samples were collected from cells, fractionated on SDS-PAGE and transferred to a nitrocellulose membrane. Immunoblots were incubated for 3 hours at room temperature with primary antibodies anti-VDR (Abcam) or anti-β-actin (Sigma). Then, membranes were incubated with anti-rabbit horseradish peroxidase-conjugated secondary antibodies (Amersham Life Sciences). Specific signal was visualized by ECL kit (Amersham Life Sciences). The protein bands (∼48 kDa for VDR and ∼42 kDa for β-actin, respectively) were quantified by Image Quant 5.0 software (Molecular Dynamics). Equal sample loading was confirmed by Ponceau S staining of the Western blot [31]. The expression levels were normalized to β-actin.

### Statistical Analysis

The normal distribution of all data was tested with Kolmogorov-Smirnov test. After confirming normal distribution, the statistical differences between different groups were analysed by one-way analysis of variance (ANOVA) for repeated measures followed by t-test. All statistical analysis was performed using statistical program SPSS 19.0 (SPSS, Chicago, IL, USA). Data are expressed as mean ± S.E.M. Differences were considered to be statistically significant at p<0.05.

## Results

### Cytokine expression in hPdLF in response to stimulation with different concentrations of *P. gingivalis* LPS and heat-killed *P. gingivalis*

**Figure 1:**
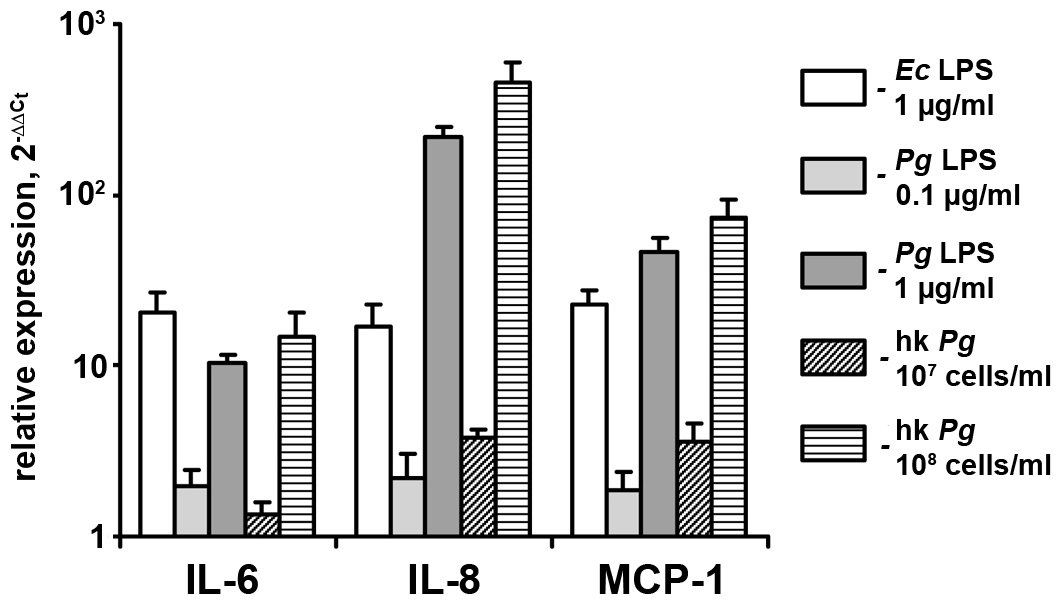
Gene expression levels of pro-inflammatory mediators in hPdLF in response to stimulation with *E. coli* LPS, *P. gingivalis* LPS, and heat-killed *P. gingivalis*. Cells were stimulated with *E. coli* LPS (1 µg/ml), *P. gingivalis* LPS (0.1–1 µg/ml), or heat-killed *P. gingivalis* (10^7^–10^8^ cells/ml) for 24 h. Gene expression levels of IL-6 (A), IL-8 (B), and MCP-1 (C) were measured using q-PCR. Y-axes represent the n-fold expression levels of target gene in relation to non-stimulated cells (control).

The response of hPdLF on the stimulation with different concentrations of *P. gingivalis* LPS (0.1–1 µg/ml) and heat-killed *P. gingivalis* (10^7^–10^8^ cells/ml) in comparison with that of well known pathogen *E. coli* LPS (1 µg/ml) is shown on the Figure 1. Gene expression levels of IL-6, IL-8, and MCP-1 significantly increased after stimulation with all stimuli. The increase in the expression levels of pro-inflammatory mediators in response to stimulation with 1 µg/ml of *P. gingivalis* LPS or 10^8^ cells/ml of heat-killed *P. gingivalis* was similar (IL-6) or markedly higher (IL-8, MCP-1) in comparison with that to *E. coli* LPS. Therefore, these concentrations of *P. gingivalis* LPS and heat-killed *P. gingivalis* were used in our further experiments.

### Effect of vitamin D_3_ on the gene expression of pro-inflammatory mediators in hPdLF

**Figure 2:**
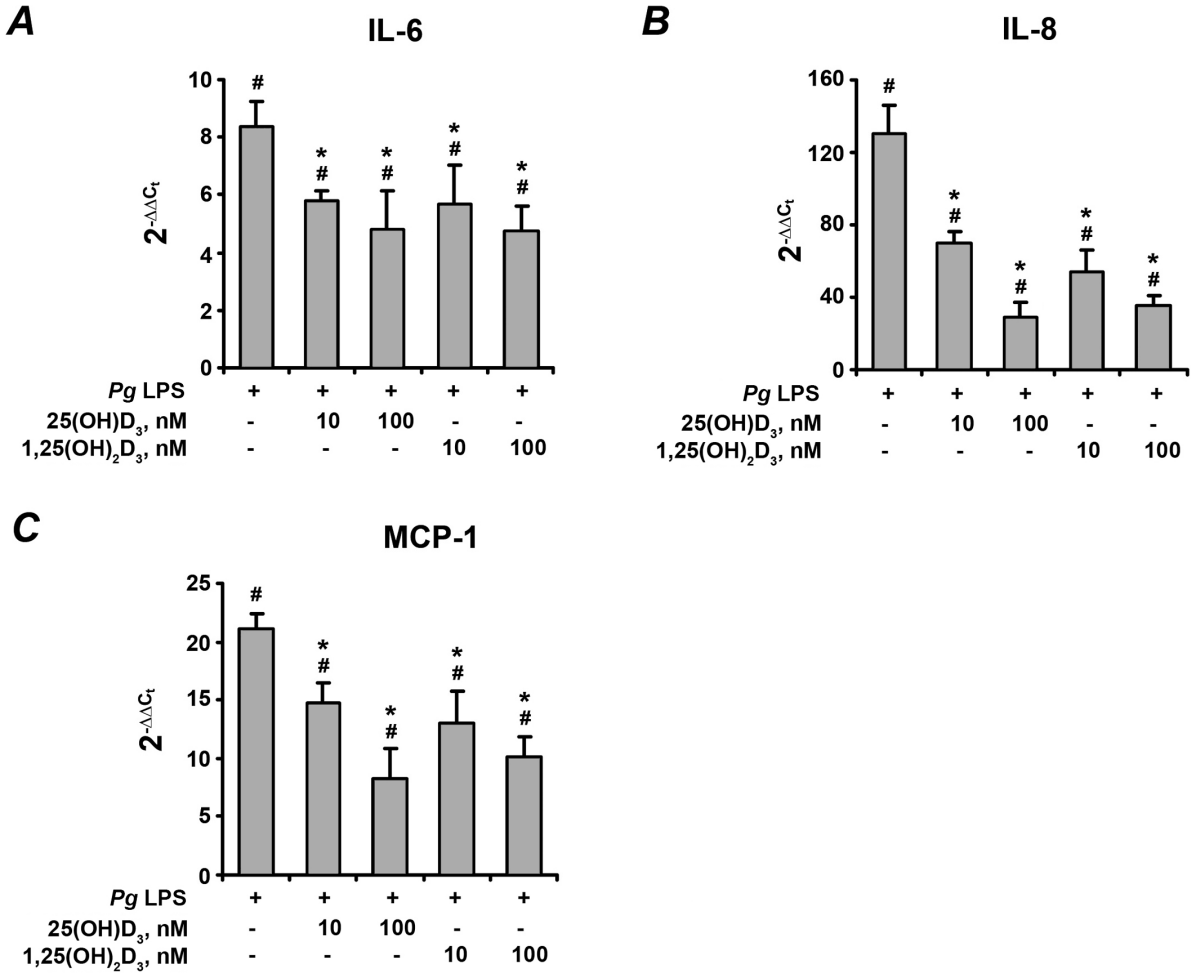
Effect of 25(OH)D_3_ and 1,25(OH)_2_D_3_ on the gene-expression levels of pro-inflammatory mediators in hPdLF in response to stimulation with *P. gingivalis* LPS. Cells were stimulated with *P. gingivalis* LPS (*Pg* LPS, 1 µg/ml) for 24 h in the presence or in the absence of different concentrations of 25(OH)D_3_ or 1,25(OH)_2_D_3_. Gene expression levels of IL-6 (A), IL-8 (B), and MCP-1 (C) were measured using q-PCR. Y-axes represent the n-fold expression levels of target gene in relation to non-stimulated cells (control). ^#^ means significantly different from control group (2^−▵▵Ct^ = 1). * means significantly different from cells stimulated with *P. gingivalis* LPS only.

**Figure 3:**
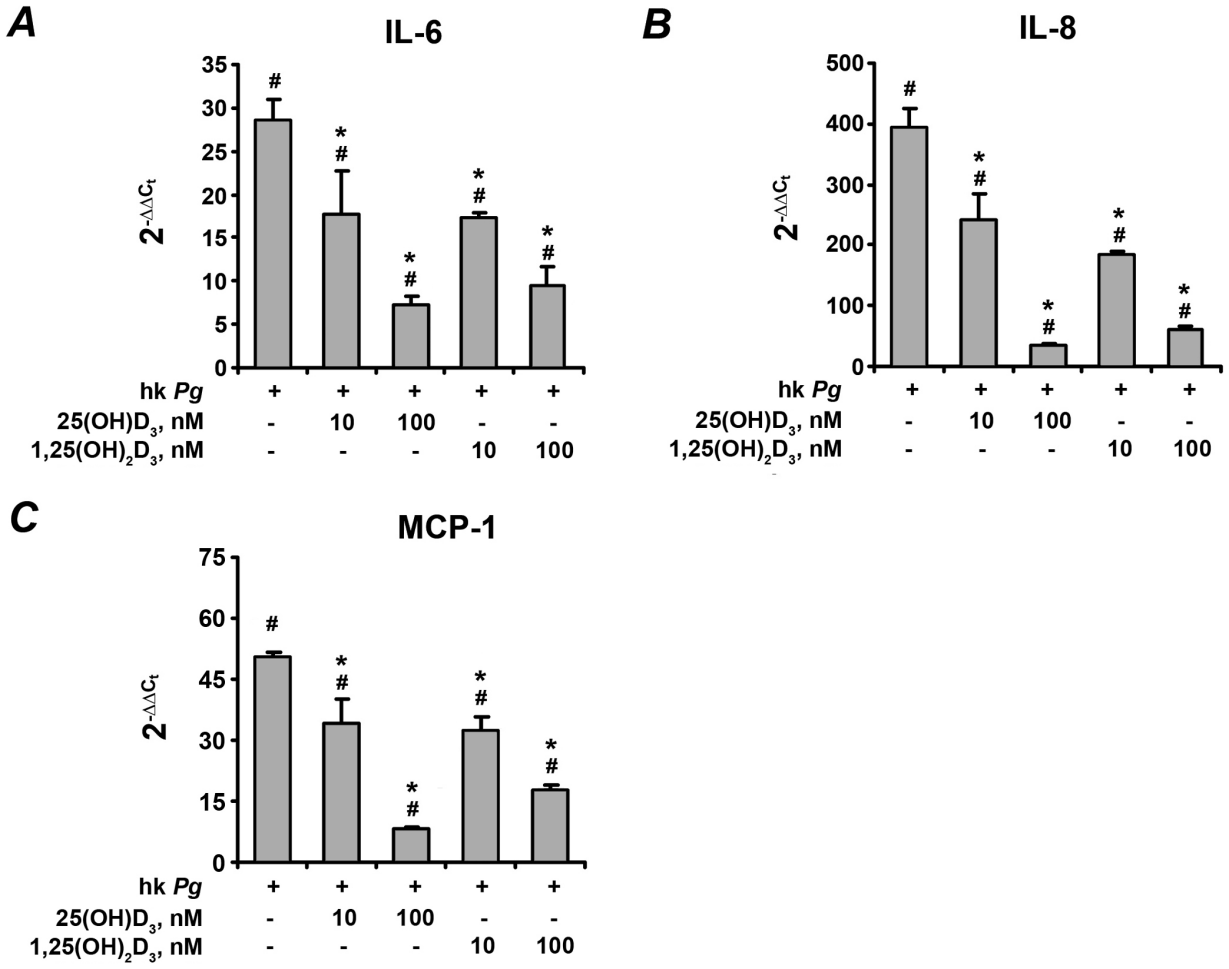
Effect of 25(OH)D_3_ and 1,25(OH)_2_D_3_ on the gene-expression levels of pro-inflammatory mediators in hPdLF in response to stimulation with heat-killed *P. gingivalis*. Cells were stimulated with heat-killed *P. gingivalis* (hk *Pg*, 10^8^ cells/ml) for 24 h in the presence or in the absence of different concentrations of 25(OH)D_3_ or 1,25(OH)_2_D_3_. Gene expression levels of IL-6 (A), IL-8 (B), and MCP-1 (C) were measured using q-PCR. Y-axes represent the n-fold expression levels of target gene in relation to non-stimulated cells (control). ^#^ means significantly different from control group (2^−δδCt^ = 1). * means significantly different from cells stimulated with heat-killed *P. gingivalis* only.

The effect of 25(OH)D_3_ and 1,25(OH)_2_D_3_ on the gene expression levels of pro-inflammatory mediators IL-6, IL-8, and MCP-1 in hPdLF in response to stimulation with *P. gingivalis* LPS and heat-killed *P. gingivalis* is shown on the Figures 2 and 3, respectively. In commercially available cells, the expression levels of all pro-inflammatory mediators were significantly increased in response to stimulation with either *P. gingivalis* LPS or heat-killed *P. gingivalis*. Both 25(OH)D_3_ and 1,25(OH)_2_D_3_ at concentrations of 10–100 nM induced a dose-dependent decrease in the *P. gingivalis* LPS- and heat-killed *P. gingivalis*-induced gene-expression levels of IL-6, IL-8, and MCP-1 (p<0.05).

### Effect of vitamin D_3_ on cytokines production by hPdLF in vitro

**Figure 4:**
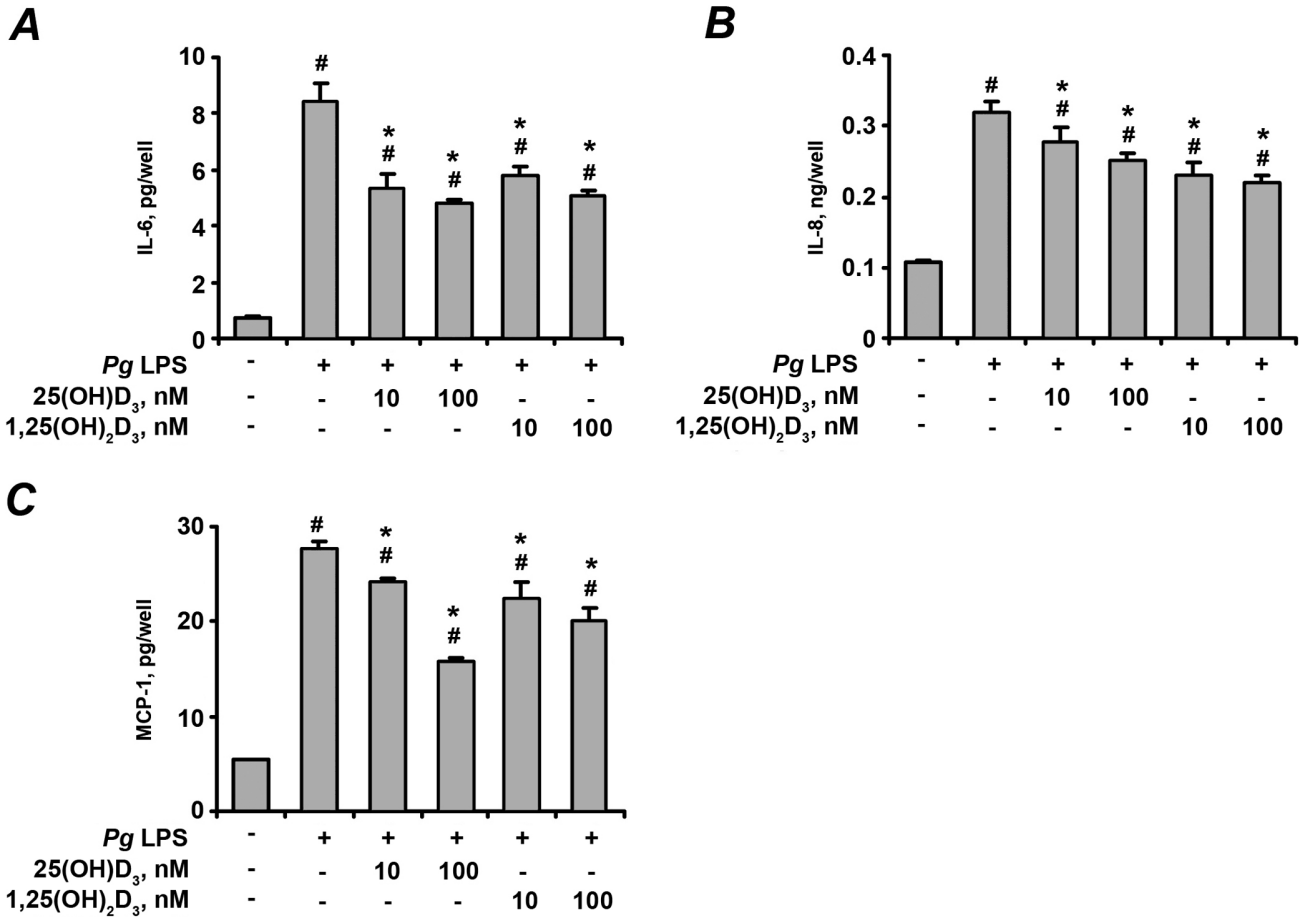
Effect of 25(OH)D_3_ and 1,25(OH)_2_D_3_ on the production of pro-inflammatory mediators by hPdLF in response to stimulation with *P. gingivalis* LPS. Cells were stimulated with *P. gingivalis* LPS (*Pg* LPS, 1 µg/ml) for 24 h in the presence or in the absence of different concentrations of 25(OH)D_3_ or 1,25(OH)_2_D_3_. The levels of IL-6 (A), IL-8 (B), and MCP-1 (C) were measured in cell supernatants using ELISA. ^#^ means significantly different from control group (non stimulated cells). * means significantly different from group stimulated with *P.gingivalis* LPS only

**Figure 5:**
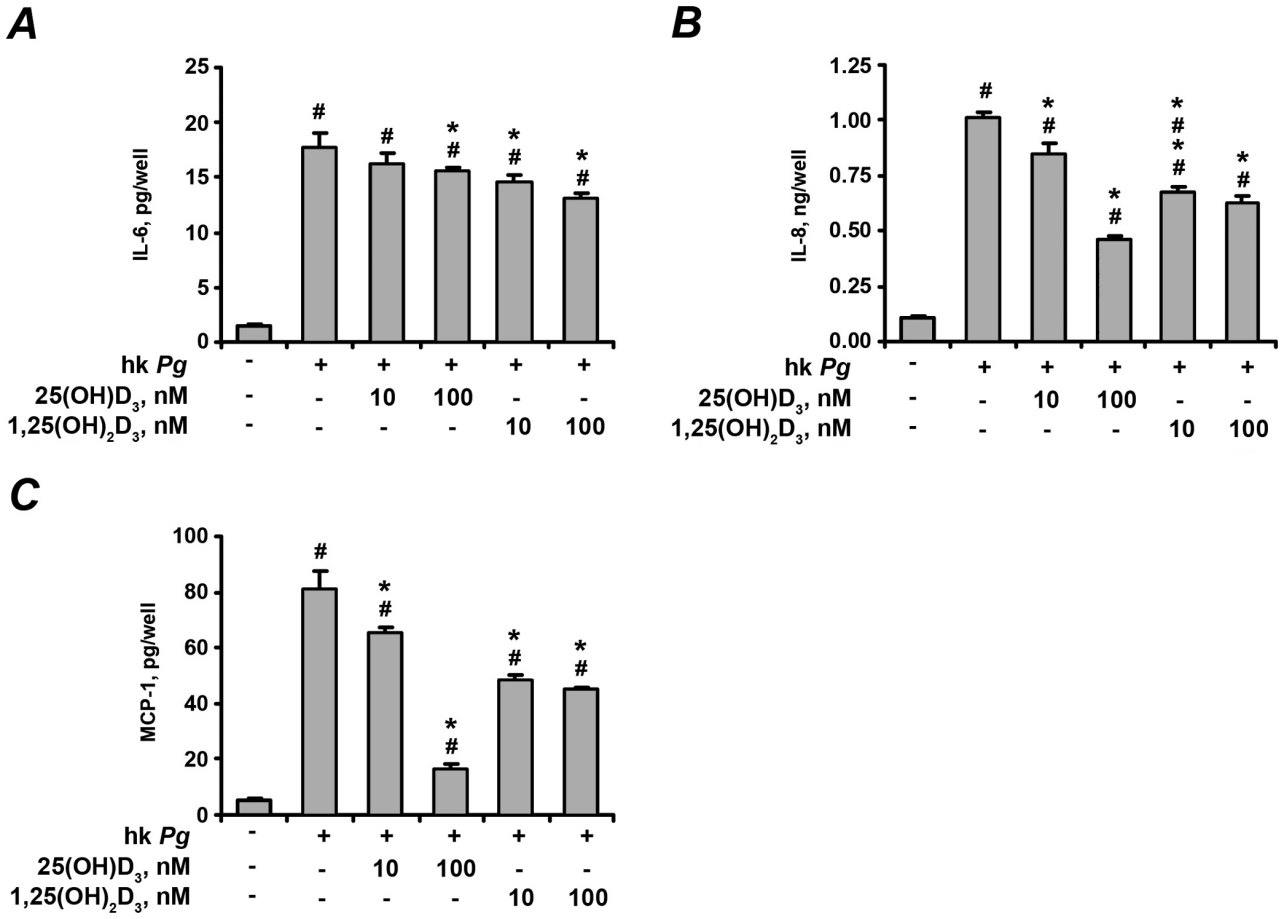
Effect of 25(OH)D_3_ and 1,25(OH)_2_D_3_ on the production of pro-inflammatory mediators by hPdLF in response to stimulation with heat-killed *P. gingivalis*. Cells were stimulated with heat-killed *P. gingivalis* (hk *Pg*, 10^8^ cells/ml) for 24 h in the presence or in the absence of different concentrations of 25(OH)D_3_ or 1,25(OH)_2_D_3_. The levels of IL-6 (A), IL-8 (B), and MCP-1 (C) were measured in cell supernatants using ELISA. ^#^ means significantly different from control group (non-stimulated cells). * means significantly different from group stimulated with heat-killed *P.gingivalis* only

The effect of 25(OH)D_3_ and 1,25(OH)_2_D_3_ on the protein content of IL-6, IL-8, and MCP-1 in conditioned media after stimulation of hPdLF cells with *P. gingivalis* LPS and heat-killed *P. gingivalis* is shown on the Figures 4 and 5, respectively. The soluble protein levels of these pro-inflammatory mediators in conditioned media were significantly increased after stimulation with either *P. gingivalis* LPS (Figure 4) or heat-killed *P. gingivalis* (Figure 5). 25(OH)D_3_ and 1,25(OH)_2_D_3_ at concentrations of 10–100 nM induced dose-dependent decrease in the *P. gingivalis* LPS- and heat-killed *P. gingivalis*-induced production of IL-6, IL-8, and MCP-1.

### Effect of vitamin D_3_ on expression of pro-inflammatory mediators in hPdLF transfected with either VDR siRNA or non-silencing control siRNA

**Figure 6:**
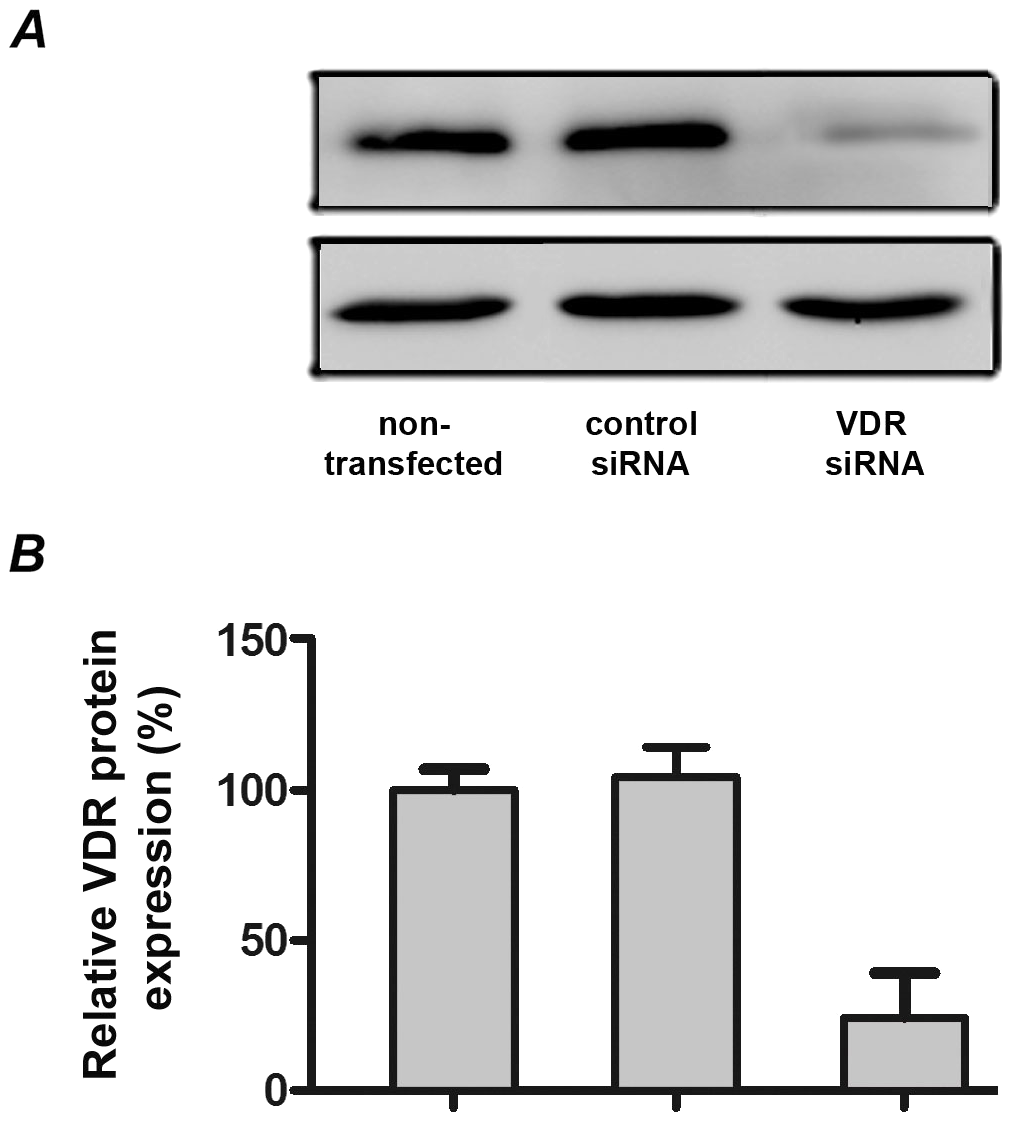
Western blot of VDR in hPdLF after transfection with VDR siRNA. (A) Original western-blots of hPdLF. Protein samples were collected from cells, and the expression of VDR and β-actin was detected using specific antibodies. (B) Quantification of western blot analysis. The protein bands of VDR and β-actin were quantified by Image Quant 5.0 software (Molecular Dynamics). The expression levels were normalized to β-actin.

**Figure 7:**
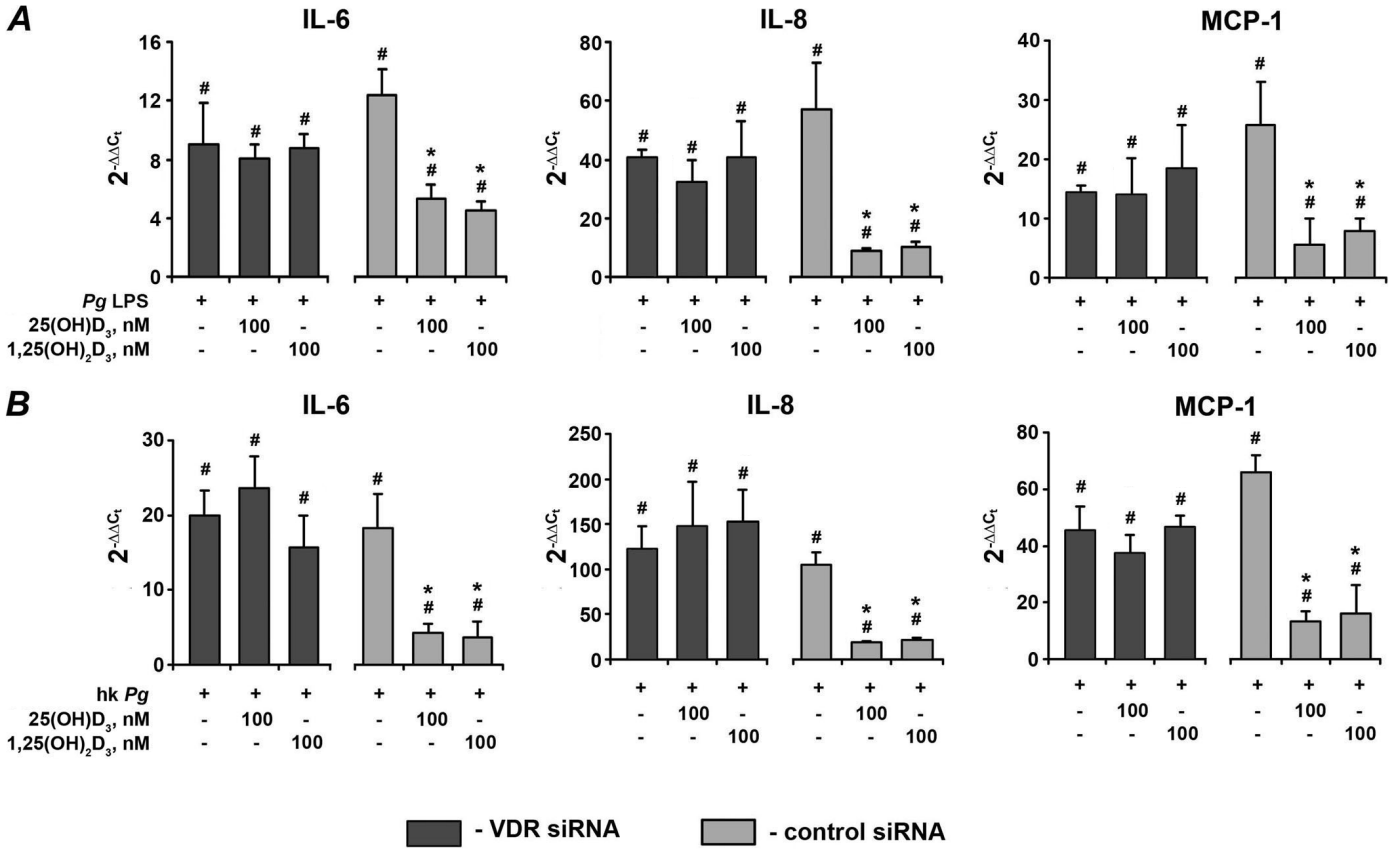
Effect of 25(OH)D_3_ and 1,25(OH)_2_D_3_ on the gene-expression levels of pro-inflammatory mediators in hPdLF with silenced VDR in response to stimulation with *P. gingivalis* LPS or heat-killed *P. gingivalis*. Gene expression levels of IL-6, IL-8, and MCP-1 were measured using q-PCR in hPdLF after transfection with either VDR siRNA or control siRNA and stimulation with *P. gingivalis* LPS (A, *Pg* LPS, 1 µg/ml) or heat-killed *P. gingivalis* (B, hk *Pg*, 10^8^ cells/ml) in the presence or in the absence of 25(OH)D_3_ or 1,25(OH)_2_D_3_. Y-axes represent the n-fold expression levels of target gene in relation to non-stimulated cells. ^#^ means significantly different from control group (2^−▵▵Ct^ = 1). * means significantly different from cells stimulated with heat-killed *P. gingivalis* LPS or heat-killed *P. gingivalis* only.

**Figure 8:**
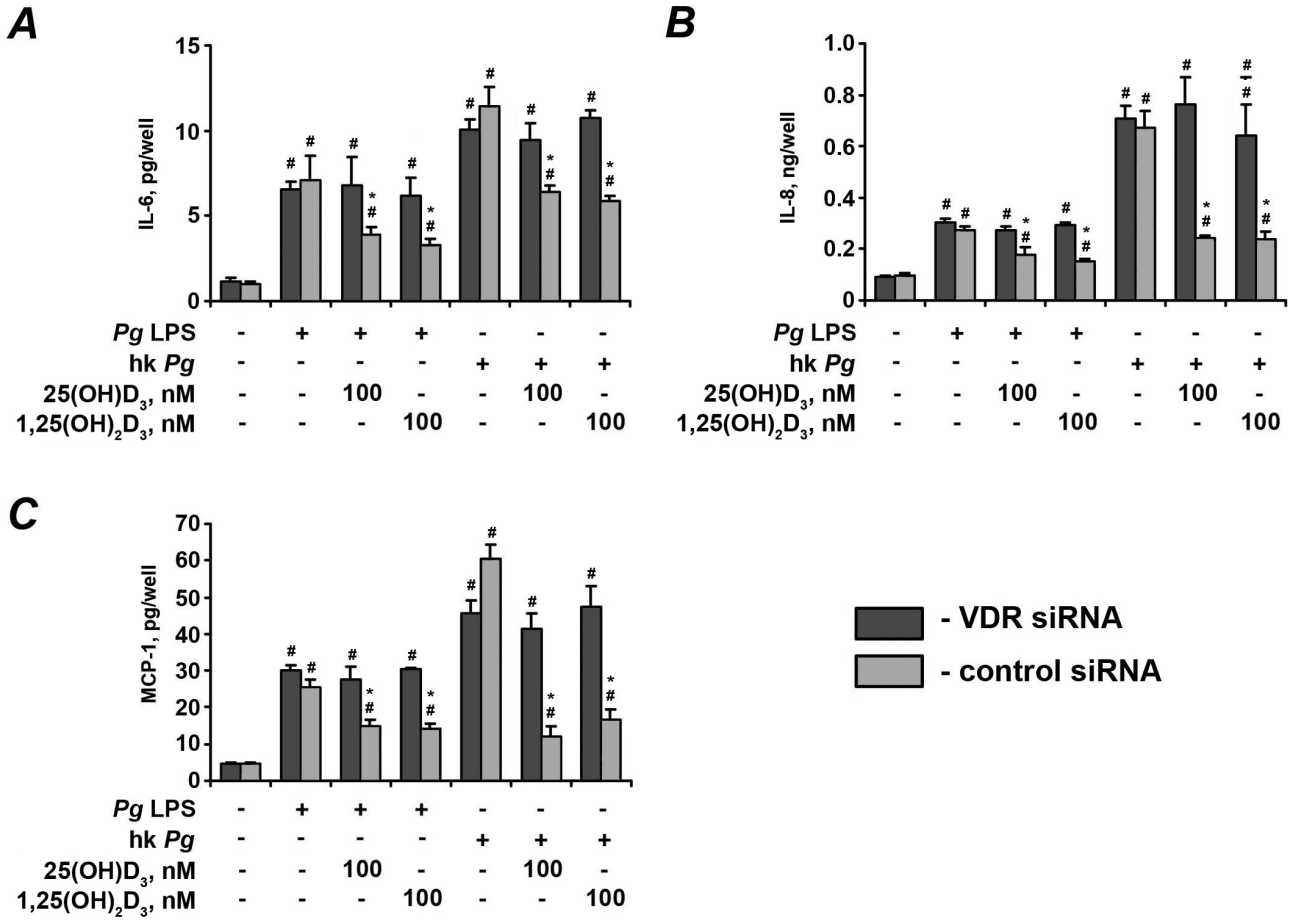
Effect of 25(OH)D_3_ and 1,25(OH)_2_D_3_ on the production of pro-inflammatory mediators by hPdLF with silenced VDR. Cells were transfected with either VDR siRNA or control siRNA and stimulated with *P. gingivalis* LPS (*Pg* LPS, 1 µg/ml) or heat-killed *P. gingivalis* (hk *Pg*, 10^8^ cells/ml) for 24 h in the presence or in the absence of 25(OH)D_3_ or 1,25(OH)_2_D_3_. ^#^ means significantly different from control group (2^−▵▵Ct^ = 1). * means significantly different from cells stimulated with either *P. gingivalis* LPS or heat-killed *P. gingivalis* only

Figure 5 shows the effect of expression of VDR protein in hPdLF after transfection with either VDR siRNA or control siRNA measured by Western blot. Transfection of hPdLF with VDR siRNA resulted in significant decrease of VDR protein expression. As measured by densitometry, transfected cells expressed about 20% of VDR protein compared to non-transfected cells. Transfection of hPdLF with control siRNA did not influence VDR expression. The effect of 25(OH)D_3_ and 1,25(OH)_2_D_3_ on the gene expression levels of pro-inflammatory mediators in response to stimulation with *P. gingivalis* LPS and heat-killed *P. gingivalis* in hPdLF transfected with VDR siRNA or control siRNA is shown on the Figures 6 and 7, respectively. The protein content of IL-6, IL-8, and MCP-1 in conditioned media is shown on the Figure 8. In hPdLF transfected with VDR siRNA neither 25(OH)D_3_ nor 1,25(OH)_2_D_3_ were able to diminish the response to *P. gingivalis* LPS or heat-killed *P. gingivalis*. This was true for both gene expression levels of IL-6, IL-8, and MCP-1 as well as for their content in conditioned media. In hPdLF transfected with control siRNA, both 25(OH)D_3_ and 1,25(OH)_2_D_3_ diminished the production of pro-inflammatory mediators in response to stimulation with *P. gingivalis* LPS or heat-killed *P. gingivalis*.

### Effect of vitamin D_3_ on cytokines expression in primary hPdLC isolated from healthy individuals

**Figure 9:**
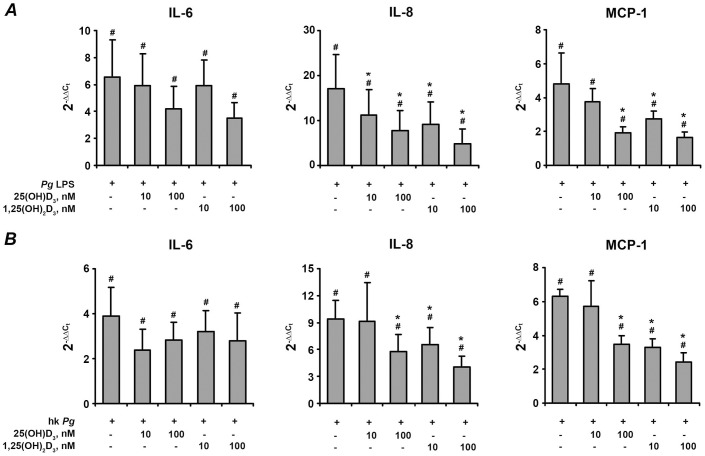
Effect of 25(OH)D_3_ and 1,25(OH)_2_D_3_ on the gene-expression levels of pro-inflammatory mediators in primary hPdLC in response to stimulation with *P. gingivalis* LPS or heat-killed *P. gingivalis*. Cells were stimulated with *P. gingivalis* LPS (*Pg* LPS, 1 µg/ml) or heat-killed *P. gingivalis* (hk *Pg*, 10^8^ cells/ml) for 24 h in the presence or in the absence of different concentrations of 25(OH)D_3_ or 1,25(OH)_2_D_3_. Gene expression levels of IL-6 (A), IL-8 (B), and MCP-1 (C) were measured using q-PCR. Y-axes represent the n-fold expression levels of target gene in relation to non-stimulated cells (control). Data are presented as mean±SEM of six different donors. ^#^ means significantly different from control group (2^−▵▵Ct^ = 1). * means significantly different from cells stimulated with *P. gingivalis* LPS or heat-killed *P. gingivalis* only.

**Figure 10:**
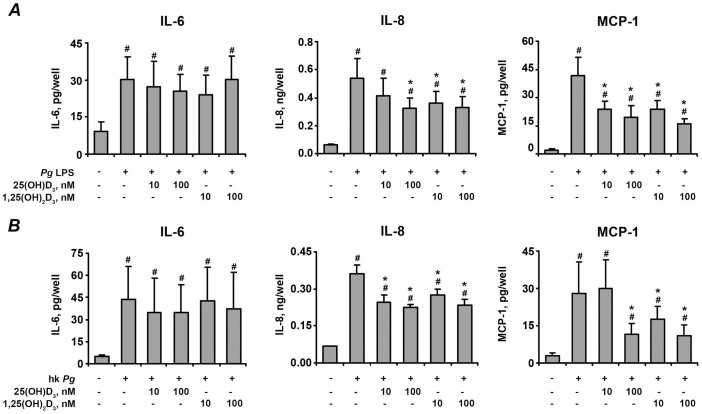
Effect of 25(OH)D_3_ and 1,25(OH)_2_D_3_ on the production of pro-inflammatory mediators by primary hPdLC in response to stimulation with *P. gingivalis* LPS or heat-killed *P. gingivalis*. Cells were stimulated with *P. gingivalis* LPS (*Pg* LPS, 1 µg/ml) or heat-killed *P. gingivalis* (hk *Pg*, 10^8^ cells/ml) for 24 h in the presence or in the absence of different concentrations of 25(OH)D_3_ or 1,25(OH)_2_D_3_. The levels of IL-6 (A), IL-8 (B), and MCP-1 (C) were measured in cell supernatants using ELISA. ^#^ means significantly different from control group (non stimulated cells). * means significantly different from group stimulated with *P.gingivalis* LPS or heat-killed *P. gingivalis* only.

The effect of 25(OH)D_3_ and 1,25(OH)_2_D_3_ on the gene expression levels of IL-6, IL-8, and MCP-1 in primary hPdLC after stimulation with *P. gingivalis* LPS and heat-killed *P. gingivalis* is shown on the Figure 9. The content of corresponding cytokines in the conditioned media is presented on the Figure 10. Similarly to hPdLF, in primary hPdLC, both 25(OH)D_3_ and 1,25(OH)_2_D_3_ induced a dose-dependent decrease in the *P. gingivalis* LPS- and heat-killed *P. gingivalis*-stimulated expression of IL-8 and MCP-1. Both forms of vitamin D_3_ tended to diminish IL-6 production by primary hPdLC but in contrast to hPdLF this effect was not statistically significant.

## Discussion

In the present study we investigated the effect of two different forms of vitamin D_3_ 25(OH)D_3_ and 1,25(OH)_2_D_3_, on the production of pro-inflammatory mediators by cells of human periodontal ligament in response to stimulation with *P. gingivalis* LPS or heat-killed *P. gingivalis*. We focused on the measurements of the expression of IL-6, IL-8, and MCP-1, which are produced by periodontal ligament cells and are thought to play an important role in the progression of periodontal disease. IL-6 is a pro-inflammatory cytokine, which plays a key role in acute inflammation phase and promotes bone resorption [32], [33]. IL-8 and MCP-1 are chemoattractant, which induce migration of neutrophils and monocytes, respectively, to the inflammation site and promote the development of acute inflammation [34], [35].

The main observation of the present study is that the production of pro-inflammatory cytokines by cells of periodontal ligament is inhibited by 25(OH)D_3_, which is the main form of vitamin D_3_ circulating in the blood and is commonly used for determination of vitamin D_3_ status. The effect of 25(OH)D_3_ was qualitatively similar to that of biologically active 1,25(OH)_2_D_3_. The optimal serum levels of 25(OH)D_3_ are thought to be about 70–100 nM [36] and these levels are similar to those used in our study. 25(OH)D_3_ is present in high amount in the gingival crevicular fluid, which is in direct proximity to periodontal ligament and its levels are increased in periodontal disease [14]. Therefore the effect of 25(OH)D_3_ on pro-inflammatory cytokine production observed in our study is physiologically relevant. Previous studies show that hPDLCs might locally convert vitamin D_3_ into 25(OH)D_3_ and subsequently into 1,25(OH)_2_D_3_ and the expression of enzyme responsible for this conversion is influenced by some pro-inflammatory mediators [20], [21]. Thus, on the one hand, the inflammatory response of periodontal ligament is regulated by both 25(OH)D_3_ and 1,25(OH)_2_D_3_ and, on the other hand, several factors involved in vitamin D_3_ metabolism are also regulated by inflammatory stimuli by feedback mechanisms. Vitamin D_3_ is also known to regulate osteogenic differentiation in periodontal ligament cells [37] and thus might affect the neighbouring alveolar bone. Therefore, it is plausible that vitamin D_3_ metabolism could play an important role in the regulation of the periodontal tissue homeostasis, especially during inflammation.

The exact physiological role of vitamin D_3_ effect on inflammatory response in periodontal ligament remains to be clarified. Decreased production of pro-inflammatory mediators by vitamin D_3_ might reduce the ability of immune system to recognize and eliminate pathogenic microorganisms on the one hand, but also could represent a protective mechanism prohibiting local excessive pro-inflammatory response and tissue destruction on the other hand [38]. Noteworthy, the effects of vitamin D_3_ in oral cavity are not associated only with decreased inflammatory response. Particularly, a study on human gingival epithelial cells shows that 1,25(OH)_2_D_3_ enhance immune response, which could lead to an increase in antibacterial activity against periodontal pathogens [39]. Periodontium is a complex tissue consisting by different cells types, which might participate in the host response to periodontal pathogens [40]. Thus, inflammatory response in various cells could be differently affected by vitamin D_3_ and this might influence the balance between bacterial elimination and tissue destruction during progression of periodontal disease. This assumption is made based on the observations of *in vitro* studies and further well-designed *in vivo* animal and/or clinical studies are required to understand the role of vitamin D_3_ in periodontitis. Since vitamin D_3_ influences inflammatory response in periodontal tissue, it might be considered as a potential tool for periodontal therapy. Some studies show that systemic vitamin D_3_ supplementation might have beneficial effect on periodontal health and periodontal therapy outcome [41], [42], [43]. Our data support the idea suggested by previous study[21], that topical application of vitamin D_3_, particularly 25(OH)D_3_, could be also considered as a potential tool in periodontal therapy.

We found that IL-6 production is inhibited by and 25(OH)D_3_ and 1,25(OH)_2_D_3_ in commercially available hPdLF but not in primary hPdLC. The reasons for this discrepancy between isolated primary hPdLC and commercially available hPdLF are not entirely clear. Interestingly, a previous study on primary hPdLC also shows that biologically active 1,25(OH)_2_D_3_ inhibits IL-8 production in response to stimulation with *P. gingivalis*, but has no effect on IL-6 production [22]. One of the possible explanations of this finding could be the methodological difference in the cell isolation protocol. In the present study, similarly to study of Tang et al, primary hPdLC were isolated by tissue outgrow method, whereas commercially available hPdLF were produced by supplier's Clonetics technique. Differences in periodontal ligament cells isolation methods are known to affect some cell properties [44]. Therefore it is possible that these changes also may lead to the modifications in cellular inflammatory responses observed in the present study. Moreover age and gender of the donor subjects may also contribute to the primary hPdLC properties [45], [46].

Our data showed that the action of both 25(OH)D_3_ and 1,25(OH)_2_D_3_ is mediated by VDR, because the silencing of this protein by specific siRNA resulted in abolishment of the effects of both forms of vitamin D. Therefore, regulation of expression levels of VDR in periodontal ligament cells might be important factor influencing functional properties of periodontal tissue. VDR is known to exhibit large polymorphism, which might contribute to different infectious disease [5], [47]. Previous clinical studies link VDR polymorphism to the chronic and aggressive periodontitis [48], [49], [50]. Therefore, the possibility that VDR polymorphism contributes to the regulation of inflammatory response by vitamin D_3_ in periodontal ligament cells cannot be excluded and requires further investigations.

Summarizing, our study shows that vitamin D_3_ modulates inflammatory response in periodontal ligament cells through vitamin D_3_ receptor. This finding suggests that both 1,25(OH)_2_D_3_ and 25(OH)D_3_ might affect inflammatory processes in periodontal disease. The exact role of vitamin D_3_ pathway in the progression of periodontal disease and possible therapeutic approaches in treatment or prophylaxis of periodontitis needs to be further investigated.
